# The adaptation and evaluation of a CBT-based manual for the inpatient treatment of youth depression: a pilot study

**DOI:** 10.1186/s40814-020-00573-6

**Published:** 2020-02-24

**Authors:** Michael Frey, Kathrin Pietsch, Laura Weninger, Matthias Brockhaus, Fabian Loy, Nathalie Claus, Petra Wagenbüchler, Selina Kornbichler, Jana Kroboth, Beatrice Georgii, Inga Wermuth, Anna Kititsina, Katharina Heiser, Gerd Schulte-Körne, Belinda Platt

**Affiliations:** Department of Child and Adolescent Psychiatry, Psychosomatics and Psychotherapy, University Hospital, LMU Munich, Munich, Germany

**Keywords:** Adolescence, Depression, Inpatient treatment, Cognitive behavioral therapy, TADS manual

## Abstract

**Background:**

Although there is good evidence to support the effectiveness of cognitive behavioral therapy (CBT) for the outpatient treatment of adolescent major depressive disorder (MDD), evidence-based manuals for the inpatient setting are lacking. This pilot study sought to (i) adapt an existing CBT manual (treatment of adolescent depression; TADS) to an inpatient setting (TADS-in), (ii) test its effectiveness at symptom reduction and remission of MDD in a pre-post design, and (iii) assess the strengths and limitations of the manual via a focus-group with clinicians.

**Methods:**

Twenty nine adolescents aged 12–17 years with a primary ICD-10 diagnosis of MDD being treated as inpatients at a psychiatric clinic were included. Embedded in the regular inpatient treatment course (8 weeks), patients received 12 sessions of the TADS-in manual. Quantitative assessment of symptom reduction and remission of MDD was conducted using a non-controlled pre-post design. The quantitative results were supplemented by a focus group with participating psychotherapists.

**Results:**

Of the 29 patients included in the study at the beginning, 19 (65.5%) remained in the study at week 8. Symptoms of depression were statistically significantly lower at the end of treatment than at baseline according to self- (*d* = 1.38; mean change = 19.88; 95% CI = 12.48–27.28) and other reports (*d* = 0.64, mean change = 0.35; 95% CI = 0.08–0.62). Clinicians ratings of improvement (CGI-I) suggested that at the end of treatment, 15.8% were very much improved, 68.4% much improved, and 15.8% were minimally improved. According to diagnostic interviews with patients conducted at the end of treatment, 73.3% were in remission. The qualitative analysis showed that on the whole, the TADS-in manual is suitable for the inpatient setting. However, clinicians believed the effectiveness of TADS-in was limited by patient comorbidity and the fact that the inpatients were unable to practice incorporating techniques learnt into everyday life.

**Conclusions:**

This study is the first to adapt the TADS manual to the inpatient setting. The sample of depressed adolescents showed reduced symptomology following treatment, although these findings require replicating in a randomized controlled trial before effects can be attributed to the TADS-in manual specifically. This pilot study informs further development of the manual as well as representing an important first step in the evaluation of the inpatient treatment of adolescent depression.

The study was retrospectively registered (DRKS00017308) and received no external funding.

## Background

### Depressive disorders during adolescence

Depressive disorders during adolescence are highly prevalent, debilitating, and recurrent. The 12-month prevalence of a major depressive episode (MDE) is 11.3% in adolescents [1] with 14–25% of youths experiencing at least one episode of major depression before adulthood [2]. Major depression restricts youth health-related quality of life as severely as no other disease [3]. The typical symptoms, such as listlessness, anhedonia, insomnia, concentration problems, low self-esteem, and social withdrawal have a negative impact on educational, employment, and social development [4]. Half of young people who take their life are clinically depressed [5]. Over the lifespan around 15–20% of depressive patients end their lives by committing suicide [6]. Although children and adolescents usually recover from their first depressive episode, 30–70% will experience one or more depressive recurrences during their childhood, adolescence, and adulthood [7].

### Cognitive behavioral approaches for treatment of adolescent depression

Meta-analyses indicate that cognitive behavioral therapy (CBT) and interpersonal therapy for adolescents (IPT-A) are the most effective psychotherapeutic treatment approaches for youth depression [8, 9] and as such they are recommended as the treatment of choice for moderate to severe cases of depression in national and international guidelines [10–12]. These guidelines also recommend the combination of psychotherapy with antidepressant medication in severe cases or when patients present with suicidal tendency. CBT is recommended as therapy of first choice with the highest level of evidence (Level 1^1^). Findings for IPT-A are less consistent (Level 2) [11, 13].

CBT is based on the assumption that thoughts, emotions, and behaviors are interconnected and influence each other. The underlying assumption of CBT is that identifying and changing one’s dysfunctional thoughts (cognitions) and behaviors will have a positive impact on emotions. Common techniques in CBT for adolescence include psychoeducation, mood monitoring, journaling, goal definition, increasing pleasurable activities (behavioural activation), identification and modification of cognitive distortions (cognitive restructuring), Socratic questioning, social communication, and personal relation skills as well as improving assertiveness and problem-solving skills to reduce feelings of hopelessness.

A CBT-based manual for the treatment of adolescent depression was developed for evaluation in the “TADS” study (treatment of adolescent depression [14];). The manual includes 15 sessions of 50–60 min and is designed to be delivered in an outpatient setting over 12 weeks. Ten of the 15 sessions are mandatory and five more can be chosen from a larger selection of modules (manual available freely at http://tads.dcri.org/wp-content/uploads/2015/11/TADS_CBT.pdf). Key components of the TADS manual include achieving measurable goals, enhancing skills in a particular area identified by the adolescent themselves, psychoeducation, self-observation, social relationship and communication skills, cognitive restructuring, general problem-solving ability, and behavioural activation [9]. The TADS randomized controlled trial (RCT) evaluated the effectiveness of the TADS manual, fluoxetine, and their combination in over 320 adolescents being treated for a MDE in an outpatient setting. TADS showed benefits in long-term response, in combination with medication, and in enhanced safety of medication in terms of suicidal tendency [15, 16].

### Inpatient versus outpatient treatment of adolescent depression

CBT for adolescent depression has almost entirely been evaluated in the outpatient setting [17, 18]. The generalizability of the findings from such studies to the inpatient setting is therefore limited. Although treatment of MDE is often performed in an outpatient setting, inpatient treatment has become increasingly common in recent years. In Germany, according to guidelines, inpatient treatment is indicated if either the severity of the symptoms leads to a significant functional impairment or there is a lack of resources and a high burden of stress factors. Suicidality in patients who are unable to promise the clinician they will not harm themselves or inadequate psychosocial support is also a reason for inpatient treatment. The number of inpatient treatment cases with MDE in the under-15s age group in Germany has increased tenfold in the last two decades (2015 compared to 2000). In the age group of 15 to 20-year-olds, they increased sevenfold [19]. This trend can also be observed internationally, albeit less pronounced [1].

There are multiple differences between the inpatient and outpatient setting which mean that developing and evaluating treatment manuals tailored to the inpatient setting are necessary. Firstly, whereas a key assumption of CBT treatment in the outpatient setting is that patients complete homework exercises in which they integrate new coping strategies into their everyday life, this may be made more difficult in the inpatient setting, where they are removed from their everyday life and have less control about how their day is structured. On the other hand, the highly structured inpatient environment may be associated with increased behavioural activation and the availability of psychotherapists. A further difference relates to the nature of cases treated in an inpatient setting. Inpatients generally present with more severe psychopathology, a higher risk of suicide, serious self–harm or self-neglect, fewer resources, higher rates of comorbidity, and reduced psychosocial functioning compared to outpatients [11, 12]. This may impact the effectiveness of manuals developed for the outpatient setting and adaptations to existing manuals may be necessary to address specific symptoms, e.g., suicidality.

In view of the different characteristics of the patients (severity of depression, comorbidity, suicidality) and the differences between the outpatient and inpatient settings, the question arises as to whether a CBT-manual, which has demonstrated its efficacy for the outpatient setting, can be transferred to the inpatient setting.

### The current study

The goal of this mixed-methods pilot study was to form a basis for a large-scale RCT of the effectiveness of CBT in the individual inpatient treatment of adolescent depression. The first specific aim was to adapt an existing CBT treatment manual (TADS) for the inpatient setting (TADS-in). The TADS manual was chosen since it integrates all the core elements of evidence-based CBT manuals and it has been successfully evaluated in the outpatient treatment of youth depression. The second aim was to conduct a pre-post (non-controlled) pilot study to test the hypothesis that the TADS-in manual is associated with (I) a reduction and (II) remission in depressive symptoms in youth with a diagnosis of depression. The quantitative evaluation also served to explore the suitability of various outcome measures in assessing the effectiveness of the manual in a further RCT. The third aim was to explore therapists’ views on the strengths and difficulties of using the manual in an inpatient setting. This was achieved by conducting a focus group with therapists who had used the manual.

## Methods

The study was retrospectively registered (DRKS00017308), and the protocol published.

### Design and participants

In a mixed-methods design, we combined a non-randomized (pre-post) study of the TADS-in treatment manual with a qualitative evaluation of therapists’ experiences in using the manual. Participants were adolescent inpatients in the Department of Child and Adolescent Psychiatry, University Hospital Munich. Inclusion criterion was age of 12 to 17 years (inclusive), ICD-10 diagnosis of MDD (F32 or F33), and normal cognitive functioning (IQ of 85 or higher). Patients with autism spectrum disorder, bipolar or psychotic disorders, personality disorder, or insufficient knowledge of the German language were excluded. Psychiatric diagnoses according to ICD-10 were established using the Diagnostic Interview of Mental Disorders in Children and Adolescents (Kinder-DIPS) [20]. The Kinder-DIPS is a well-established German diagnostic interview which can be completed with the patient themselves or their parent. For this study, we used the patient version, which has retest reliabilities of Cohen’s kappa for MDD = .94 [.85; 1.00] [21]. The Kinder-DIPS was conducted by researchers trained in using it. If cognitive ability could not easily be detected from the preliminary clinical impression, an intelligence screening was carried out using the Culture Fair Intelligence Test (CFT-20-R [22];.

We conducted a sample-size calculation using G*Power [23] based on a Wilcoxon signed-rank (matched pairs) test (two-tailed) and the effect size found for the CBT-only condition on depressive symptoms in the original TADS study (ES = 1.53^2^; March et al. [15]). We assumed power of 0.8 and an alpha error probability of 0.05 which required us to recruit at least six patients to the study to find the desired effect. Because we were unsure how much dropout and missing data to expect and because we wanted to have a sufficient number of patients to have a reasonable level of confidence about the generalizability of the findings, we aimed to recruit patients until we had included 30. Between Spring 2013 and Summer 2017, all patients who met the inclusion criterion were invited to participate (*n* = 51). Of the 51 patients invited, 29 (56.9%) participated. A focus group with six therapists who had delivered the intervention and a study nurse involved in recruitment of patients was conducted in Summer 2017 (see “Focus Group”). Table 1 summarizes the demographic characteristics and baseline diagnoses of the 29 participants included in the study.

**Table 1:** Participant characteristics and baseline diagnoses

	Number of participants/mean (SD)
Gender M/F	9/20
Age in years (mean ± SD)	14.6 (1.69)
Depression severity	
Mild	5
Moderate	12
Severe	12
Comorbidity	
Separation anxiety	3
Specific phobias	3
Test anxiety	2
Social phobias	10
Agoraphobia	1
Generalized anxiety	2
PTSD	1
ADHD	1
Conduct disorder	1
Eating disorder	3

### Intervention

**Fig. 1:**
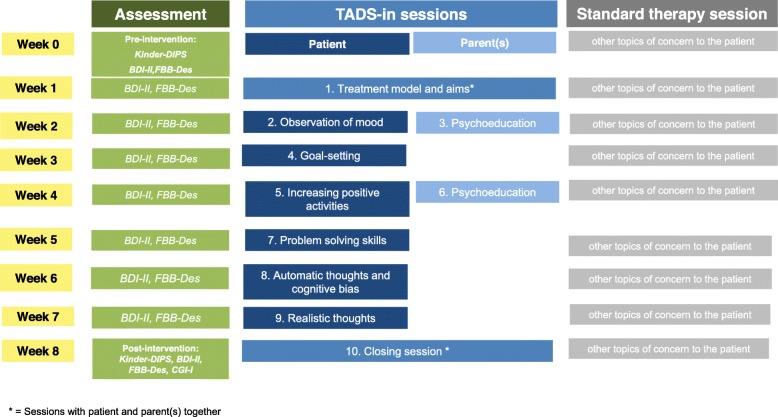
Overview of the TADS-in structure and contents

An overview of the “TADS-in” manual is provided in Fig. 1 (manual available upon request). In adapting the original TADS manual for the inpatient setting (TADS-in), the number of sessions was reduced from 15 to 10 and excluded the five optional sessions for feasibility reasons. Within the ten sessions, there were six single sessions with the patient covering the following topics: psychoeducation about depression and its causes, goal-setting, mood monitoring, increasing pleasant activities, social problem-solving, and cognitive restructuring. Additionally, there were two parent-only sessions which provided psychoeducation about depression, and two conjoint parent- and adolescent-sessions, focused on addressing parent and adolescent concerns. The sessions were scheduled for 8 (rather than 12) weeks. Treatment as usual at the Department of Child and Adolescent Psychiatry, University Hospital Munich, includes two single psychotherapy sessions per week. For participants in the study, the TADS-in manual was delivered in one of them while the second session was used for the patients to discuss other concerns, e.g., conflicts with friends or family, school and problems.

Patients received treatment from trainee psychiatrists who had (*n* = 11) or had not (n = 4) begun their psychotherapy training, trainee clinical psychologists (*n* = 9), qualified psychiatrists (*n* = 3), or trainee psychotherapists (*n* = 2; undergraduate degree in educational science). The therapists had an average of 3.56 years (SD = 2.32) of experience in delivering psychotherapy. The majority of the therapists had a (cognitive) behavioral therapy background (48.3%), whereas around one third were trained/training in psychodynamic psychotherapy (34.5%) and 17.2% were training to become psychiatrists but had not yet begun their psychotherapy training. All therapists were offered specialist supervision by a consultant psychiatrist and psychotherapist concerning questions and uncertainties relating to delivering TADS-in.

### Quantitative outcome measures

An overview of the assessment instruments used in the study is provided in Table 2.

**Table 2:** Overview of assessment instruments

Domain	Assessment instrument
Diagnosis and symptom severity	Kinder-DIPS
Depressive symptoms	BDI-II (self-assessment; weekly) FBB-DES (external assessment; weeky)
Clinical Global Impression	CGI-I (clinician report)

### Symptoms of depression (self- and other report)

Our primary outcome measure was change in depressive symptoms post-intervention which we assessed via self-report and reports provided by healthcare assistants working on the ward where treatment took place.

The Beck Depression Inventory (BDI-II) was used for self-assessment [24]. The BDI–II is a 21-item, self-report instrument that measures severity of depression in adolescents and adults. Items assess symptoms corresponding to criteria for diagnosing depressive disorders listed in the DSM-IV. Response options include four increasing levels of severity. Scores for each item range from 0 to 3; the total score is the sum of all responses; 0–8 indicates no depression, 9–13 minimal depression, 14–19 mild depression, 20–28 moderate depression, and 29–63 severe depression. The internal consistency for the German version was described as around .90 and the retest reliability as .78.

To obtain an external report of symptoms, we used the FBB-DES: a depression rating scale, which is part of the Diagnostic System for Mental Disorders in Childhood and Adolescence (DISYPS-II) and based on the international classification systems ICD-10 and DSM-IV [25]. The FBB-DES consists of 42 items in total. The first 29 items describe symptoms of depression which are rated on a four-point scale from 0 (not at all true of the patient) to 3 (very true of the patient). An average score across these 29 items is generated and provides a measure of symptom severity. The internal consistency of the FBB-DES is *α* > .70. Correlations with self-assessment and diagnostic checklists for depression in clinical trials indicate a good convergent and divergent validity of the test [26]. In this study, the FBB-DES was completed by healthcare assistants working on the ward where treatment took place. The test-retest reliability of the FBB-DES was calculated using data from baseline and after the intervention. BDI-II and FBB-DES were assessed at baseline, once a week and immediately after completion of the treatment. Calculating the correlation between FFB-DES and BDI-II, we used the data at baseline.

### Clinician-rated improvement

Our secondary outcome was clinician-rated overall change in impairment after treatment and was measured using the improvement score of the Clinical Global Impression scale (CGI-I) [27]. The CGI is a widely used tool originally developed for clinical trials to provide a brief assessment of the clinician’s view of the patient’s global functioning prior to and after a pharmaceutical intervention [28, 29]. It comprises of two one-item measures (each rated on a 7-point scale) evaluating the following: (a) CGI-S: severity of psychopathology at baseline compared to other patients and (b) CGI-I: change from the beginning to end of treatment. We only used the CGI-I since we were primarily interested in symptom change. The CGI-I scale ranges from 1 = very much improved since the initiation of treatment to 7 = very much worse since the initiation of treatment.

### Depression remission

Our tertiary outcome was remission of depression post-intervention. To assess whether the depression was remittent, the “Depression” subscale of the Kinder-DIPS [20] was readministered at the end of treatment by members of the research team who had not treated the patient. Remission was defined as the patient no longer meeting ICD-10 diagnostic criteria for an MDE.

### Treatment fidelity

Treatment fidelity was assessed based on standardized checklists that therapists filled out at the end of each session (See Additional File 1 for an example; all checklists available upon request).

### Qualitative outcomes

The quantitative results were supplemented by a qualitative analysis of the strengths and weaknesses of the manual. For this purpose, the experiences of the psychotherapists who had delivered TADS-in were shared in a focus group. Seven therapists took part and the session lasted 90 min. The focus group followed an interview schedule (see Additional File 2) which was adapted from an “Experience of Therapy” interview schedule provided by Nick Midgley and covered the following contents: logistical issues associated with TADS-in (e.g., frequency of sessions), patient issues (e.g., which patients were most/least suited to the manual), content of TADS-in (which elements were particularly helpful/unhelpful), therapist issues (e.g., how much did you enjoy using the manual), and other therapy topics and concerns of patients not already discussed. The meeting was audio recorded and transcribed verbatim into word.

### Data analysis

Data were analyzed for those who remained in the study at the final assessment point (week 8). Statistical analyses on pre-post change in BDI-II scores and FBB-DES scores were conducted using a Wilcoxon related-samples signed-rank test (SPSS). Weekly assessments of the BDI-II and FBB-DES were initially planned but only completely available for one patient, meaning a more detailed analysis of change in depressive symptoms over time was not possible. Clinician-rated improvement in symptoms (CGI) and diagnostic information on the remission of depression (Kinder-DIPS, based on the diagnostic criteria of the ICD-10) are reported descriptively. To evaluate the correlation between self- and external symptoms of depressive symptoms at baseline, a Pearson correlation coefficient was calculated.

The evaluation of the focus group was performed with the software “NVivo” (QSR International Pty Ltd., 2012). An inductive thematic analysis was carried out, following the six stages of analysis recommended by Braun & Clarke [30]. The transcript was read and re-read, and major themes recorded. A coding frame was developed to facilitate coding, and final identification of themes was based on consensus discussion between members of the research team. Key themes were described and illustrated with quotes and then related to each other and back to the research question. Answers are reported below grouped around those themes, with all quotes translated into English from their German original. Since analysis relied solely on an audio (rather than video) file, general nods of agreement could not be included and so the number of people endorsing each issue may be an underestimate.

## Results

**Fig. 2:**
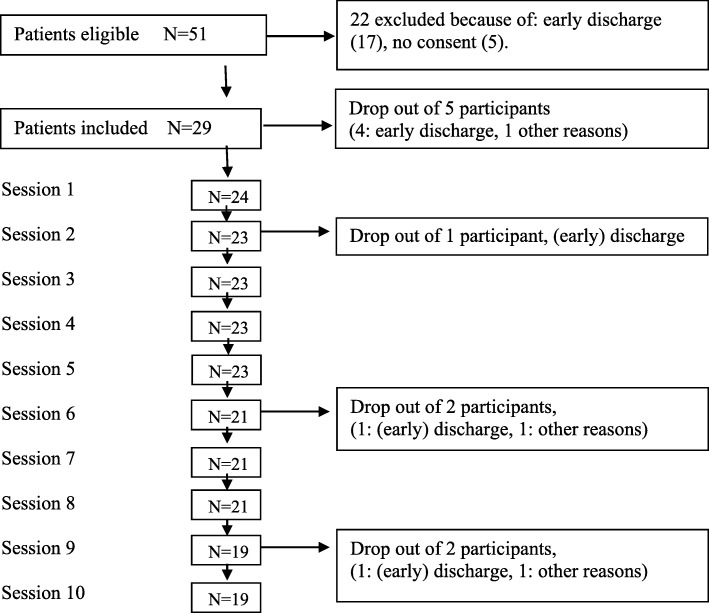
Patient flow from recruitment to intervention completion

Figure 2 provides an overview of the patient flow from entrance into the study to completion of the manual.

### Subject characteristics

The 29 participants (69.0% female) included in the study had a mean age of 14.6 years (SD = 1.6). Based on the clinical interviews (Kinder-DIPS), depression severity ranged from mild (17.2%) to moderate (41.4%) or severe (41.4%). The total raw score on the BDI-II at baseline ranged from 10 to 52, and the mean score was 33.3 (SD = 12.2). For 86.2%, it was the first depressive episode. 62.1% of participants had comorbid disorders, mostly exclusively other internalizing disorders (66.7%), especially social phobia (55.6%). 27.8% met the criteria for more than two axis one diagnoses.

### Dropout and treatment fidelity

Of the 29 subjects included in the study, 19 (65.5%) remained in the study at week 8. The main reason for leaving the study was the discharge from the inpatient setting during the study period (8/10; 80.0%). Most patients were discharged because their symptoms had reduced to a level where an inpatient stay was no longer necessary. Other patients left the study because their treatment focus had changed because of a comorbid disorder.

Of the 29 patients included in the study, data on treatment fidelity was available for 24 (82.8%). These patients had completed 8.6 of the 10 sessions on average (SD = 2.4). Fourteen patients (58.3%) had completed all 10 sessions. On average (across all sessions and all 24 participants), 81.6% of the manual contents were delivered (SD = 23.5%).

### Symptoms of depression

#### Self-report

**Fig. 3:**
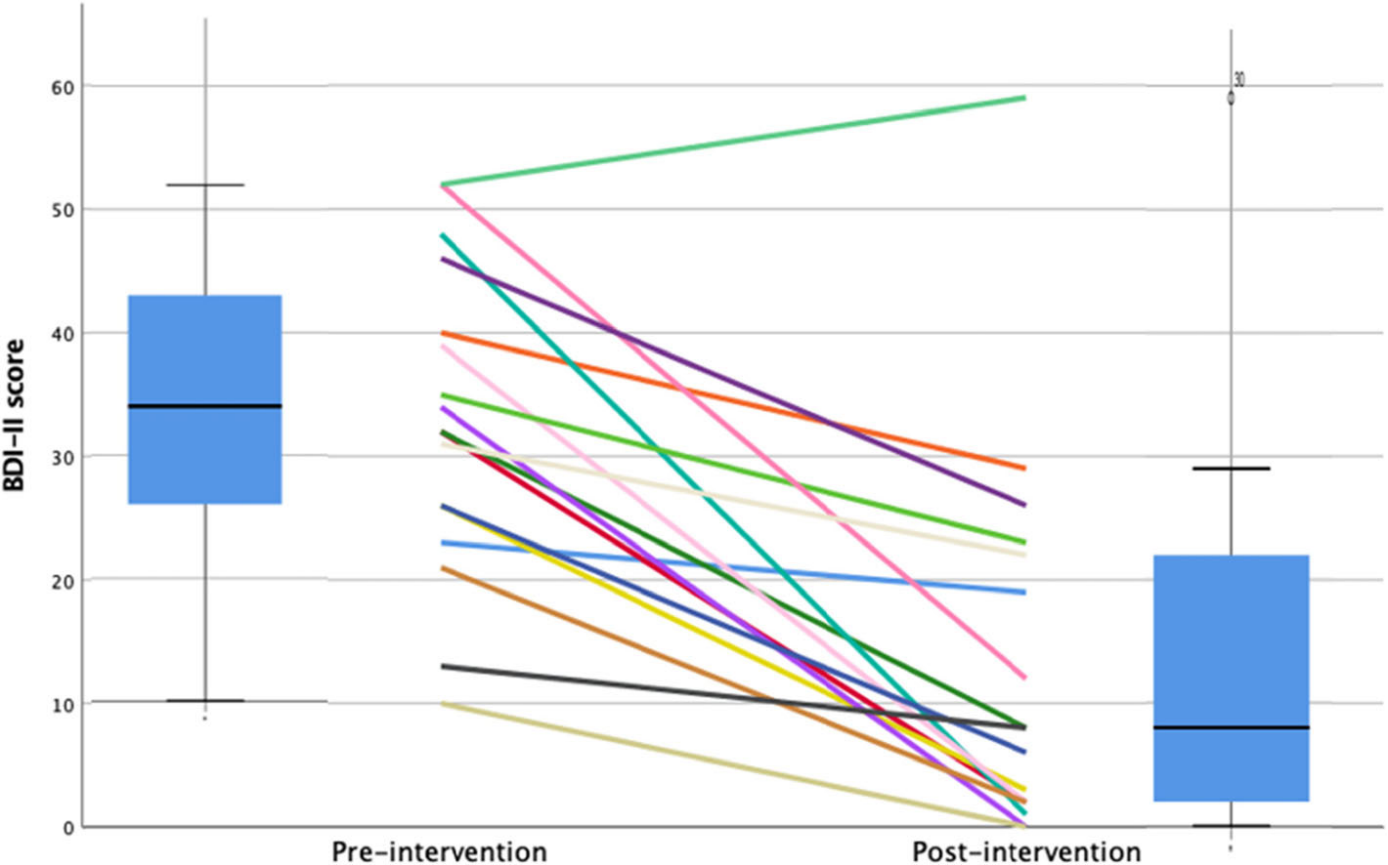
Self-report depression scores (BDI-II) from baseline to end of treatment for 17 adolescents

Baseline and post-intervention BDI-II data were available from 17 of the 19 patients who completed the intervention. The BDI-II total raw scores at the end of the treatment (median = 8.00 [0; 59]) were lower than at baseline (median = 34.00 [10; 52]); this difference was statistically significant according to the Wilcoxon Related-Samples Signed-Rank test (mean = 19.88; 95% confidence interval = 12.48–27.28). The effect size was *d* = 1.38^3^ [31]. Figure 3 illustrates BDI changes for each individual (box plot indicates median).

#### External report

**Fig. 4:**
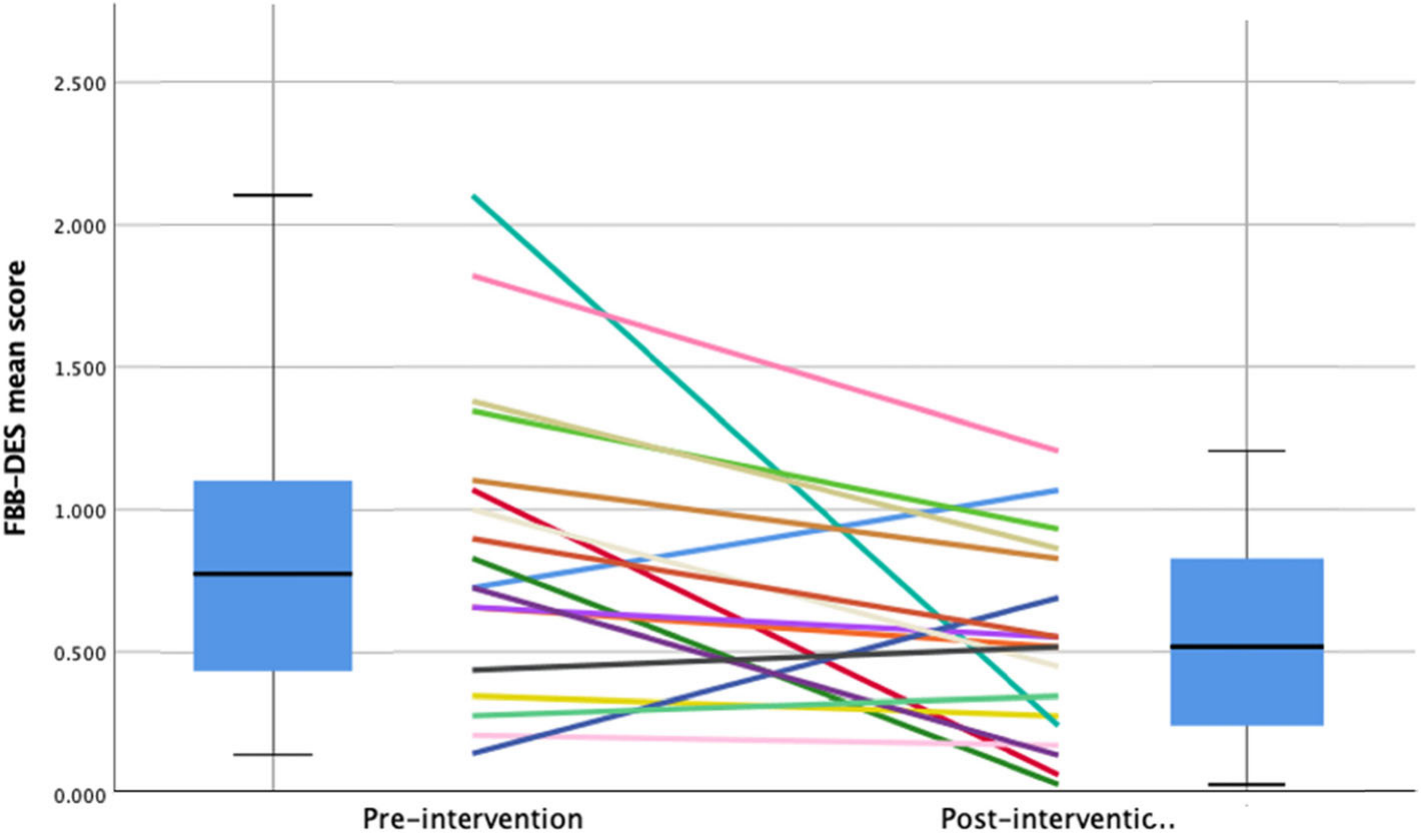
Other-report depression scores (FBB-DES) from baseline to end of treatment for 18 adolescents

Figure 4 illustrates FBB-DES changes for each individual (box plot indicates median). Baseline and post-intervention FBB-DES data were available from 18 of the 19 patients who completed the intervention.

The FBB-DES mean scores at the end of the treatment (median = 0.52; [.03; 1.21]) were lower than at baseline (median = 0.78; [.14; 2.10]). This difference was statistically significant according to the Wilcoxon related-samples signed-rank (mean = 0.35; 95% confidence interval = 0.08–0.62). The effect size was *d* = 0.64^4^ [31]. The test-retest reliability of the FBB-DES in our sample was *r* = 0.31 and *p* = 0.2. The correlation between FBB-DES and BDI-II scores at baseline was *r* = 0.28 and *p* = 0.27.

### Clinician-rated improvement

CGI-I values were available for all participants who received the intervention (*N* = 19). At the end of the TADS treatment, 15.8% of the patients were “very much improved,” 68.4% “much improved,” and 15.8% were “minimally improved.” None of the patients had worsened or remained stable.

### Remission of depression

Diagnostic data (Kinder-DIPS) upon completion of the intervention were available for 15 of the 19 participants who completed the intervention. At the end of the study, 11 of the 15 patients (73.3%) were remitted and no longer met the ICD-10 criteria for depression. Two patients who were moderately or severely depressed at the beginning of the study only met criteria for mild depression at the end of the study. One patient was unchanged (continued to meet criteria for a severe episode) and another had worsened from a moderate episode to a severe depressive episode.

### Correlation between external and self-assessment of depressive symptoms

There was no evidence of a correlation between external (FBB-DES) and self-reported (BDI-II) symptoms of depression (*r* = .28, *p* = .27).

### Qualitative analysis

The findings from the focus group are presented below according to the five overall themes which emerged (i) factors of the inpatient setting conducive and (ii) obstructive to TADS-in, (iii) inpatient factors conducive and obstructive to TADS-in, (iv) benefits of TADS-in for parents, and (v) helpful and unhelpful aspects of the TADS manual in general.

### Factors of the inpatient setting conducive to TADS-in

Therapists agreed that overall, TADS-in fitted the inpatient setting well (*n* = 2)^5^. Key components of TADS-in such as behavioural activation and implementing positive activities were identified as already being incorporated into the general psychotherapeutic approach of the inpatient setting (*n* = 3). Furthermore, as a result of the presence of healthcare assistants in the inpatient setting, behavioural activation and implementing positive activities may have been easier to implement than in the outpatient setting. For example, one therapist said:

If it’s clear [their goal is] one game per day, they might have a problem at home, they don’t know who to play what with or where. And like this [in the inpatient setting] the PED [healthcare assistant] was aware, okay, he’s supposed to play something once a day.

One therapist also pointed out that therapists typically had more time for preparation of TADS-in sessions than they might have done in an outpatient setting.

### Factors of the inpatient setting obstructive to TADS-in

The focus group identified several features of TADS-in as being difficult to implement in an inpatient setting. Firstly, patients often had very complex additional issues—more than one would expect in an outpatient setting—which needed to be addressed during treatment (*n* = 2). Therapists identified these issues to include interactions/problems with their family (*n* = 2), problems on their ward (*n* = 2), school (*n* = 2), self-harm/suicidal thoughts and behaviors (*n* = 2), sleeping problems (*n* = 1), and planning for discharge (*n* = 1). A second therapeutic session each week was designed for this purpose, but some therapists felt this was still not enough (*n* = 2). This may be why the manual was described as too dense, with too much input within not enough time (*n* = 4) and sessions often taking longer than intended (*n* = 2). Therapists made suggestions in order to avoid rushed sessions in the future: either to reduce the amount of introductory content at the beginning of the intervention (which was often already familiar to inpatients) and leave more time for more complex topics at the end (*n* = 3) or include additional sessions to spread out the same topics over a longer time frame (*n* = 1).

A second restriction was that being away from their everyday life meant that it was difficult for patients to practice techniques which would help them following discharge (*n* = 3). One therapist said:

I found that very difficult in this setting, if they want to cycle or go swimming more often, more horse riding, something like that. That just wasn’t possible. Or just meeting friends twice a week or something, that wasn’t possible, either.

Clinicians also felt that the fact that children were staying away from home meant that parents also lacked the time to implement changes (*n* = 2).

A third restriction of the inpatient setting identified by therapists was logistical issues such as discharge planning and restricted time resources (*n* = 2).

Finally, the inpatient setting meant that patients shared the contents and topics of TADS-in sessions with each other, which sometimes had the negative effect that there was confusion about what topics to expect each week when some patients had started the intervention earlier than others.

### Patient factors conducive and obstructive to TADS-in

Therapists described TADS-in as making sense particularly for patients (i) with a lower level of social and educational functioning who might be more willing to try something new (*n* = 3), (ii) with a need (*n* = 3) or preference (*n* = 2) for working with a clear structure, and/or (iii) who were reluctant to open up to the therapist of their own accord (*n* = 5).

Patient characteristics that made TADS-in more challenging or less effective were (i) high levels of impairment (especially in their cognitive function) and/or comorbidity (e.g., school absence, *n* = 2; anorexia nervosa, *n* = 1) which were associated with difficulty concentrating (*n* = 4), (ii) when their disorder profile changed too much over the course of their therapy (*n* = 1), and/or (iii) when they had a clear idea of the topics they wanted to cover in psychotherapy (*n* = 2).

Another factor that was considered before offering TADS-in treatment was the patient’s previous experiences of psychotherapy and resulting expectations (*n* = 2); previous experiences could mean that patients were either irritated by the rigid schedule of the TADS-in manual or already too familiar with typical CBT contents to benefit further. Additionally, TADS-in was not the first choice of treatment for patients who lacked motivation for therapy and change (*n* = 2), although this aspect was not specific to TADS-in, but applied to psychotherapy in general.

### Benefits of TADS-in for parents

Compared to usual inpatient treatment, therapists felt that TADS-in improved parents’ interactions with their children. Therapists felt the structure and concrete contents of TADS-in made therapy more transparent for parents (*n* = 3), whereas conversations with parents were otherwise more concerned with current problems at home or a goal-oriented assessment of the child’s therapy progress. The shared knowledge provided a framework for talking about therapy and depression and encouraged more active parental participation, which led to more openness (*n* = 2). One therapist also pointed out that TADS encouraged parents to attend appointments together even if they were separated, which was often a problem with the usual inpatient treatment. Despite these positive aspects, clinicians did report that the TADS-in sessions with parents felt too dense (*n* = 2). Therapists also pointed out that TADS-in was not better than usual treatment for all families (*n* = 1).

### Helpful and unhelpful aspects of the TADS manual in general

Therapists also commented on aspects of the TADS manual more generally (i.e., not specific to the inpatient adaptation). Sessions on cognitive restructuring and the relationship between thoughts and feelings (*n* = 3) were deemed helpful to the extent that clinicians used material from them with patients not being treated with TADS-in. The mood thermometer was deemed a useful tool for easier self-evaluation that was used reliably by patients even if other homework was neglected (*n* = 2). The concrete stories, e.g., the tennis trainer, were deemed helpful for illustration purposes (*n* = 2).

Therapists only made one specific suggestion for changes to the manual: two would have preferred a different term for homework assignments since “homework” had a negative connotation for patients.

## Discussion

### Summary of findings

The overarching goal of this pilot study was to adapt an individual outpatient CBT manual for the individual inpatient treatment of depression in adolescents. To investigate feasibility and effectiveness, this mixed-methods study sought to (1) adapt an existing treatment manual (TADS; March et al. [15]) for the inpatient setting (TADS-in), (2) conduct a pre-post (non-controlled) pilot study to test the hypothesis that the TADS-in manual is associated with (I) a reduction in symptoms of depression and (II) a remission of a depressive episode, and (3) explore therapists’ views on the strengths and difficulties of using the manual in an inpatient setting. Quantitative analysis showed a significant reduction in depressive symptoms according to self- and clinician ratings. External ratings provided by healthcare assistants also showed a reduction in symptom severity, albeit it of a smaller effect size. A diagnostic interview (Kinder-DIPS) at the end of the treatment showed that 73.3% of the patients no longer met diagnostic criteria for an episode of depression. Findings from the focus group suggested that the TADS-in manual had numerous strengths and limitations. On the one hand, key contents of the TADS manual were enhanced by the inpatient setting, e.g., behavioural activation and implementing more positive activities. On the other hand, the inpatient setting made it difficult for patients (and their parents) to transfer therapy contents into everyday life. We first discuss the findings from the quantitative data analysis and their implications for future studies, before considering the outcome of the focus group and potential modifications which could be made to improve the TADS-in manual.

### Interpretation of quantitative findings

In order to appropriately interpret the findings of the pre-post assessment, it is important to first assess treatment fidelity (whether the intervention was really delivered as intended). Data on treatment fidelity was available for 24 of the 29 participants included in the study and included five patients who dropped out of the study prior to completing the intervention. On average, 8.6 of the 10 sessions (SD = 2.41) were completed and 14 patients (58.3%) had completed all 10 sessions. Considering just the 19 patients who remained at week 8 fidelity was even higher: 9.8 of the 10 sessions (SD = 0.42) were completed and 15 (78.9%) had completed all 10 sessions. Together, this suggests relatively high treatment fidelity. Homework, however, was completed in less than half the cases (45.8%) despite the inpatient setting, which through the presence of staff members we had expected to be more conducive to homework than the outpatient setting. The session most frequently omitted was the one with the parents (session 6) which raises questions about the compliance of the parents. This could be due to the fact that hospitalization initially relieves the burden on the family system and primarily attributes the need for change to the patient.

In the pre-post comparison of the 19 patients who provided data at both time points, there was a significant decrease in self-reported symptoms of depression with a very “large” effect size (ES = 1.38). The effect is comparable to the results of CBT alone in the TADS study (ES = 1.53; March et al. [15]). This pattern of findings was similar across patient, clinician, and diagnostic measures, supporting the robustness of the finding. Nevertheless, we found a smaller reduction of symptoms according to ratings provided by healthcare assistants (*d* = 0.64) which represents a “medium” effect size [31]. This may reflect the fact that for each patient, different healthcare assistants completed the assessments at baseline and post-treatment. Indeed, the test-retest reliability of the FBB-DES in our sample was low (*r* = 0.31, *p* = 0.2). Furthermore, there was a low correlation between FBB-DES and BDI-II scores at baseline (*r* = 0.28, *p* = 0.27). Although a controlled trial is needed to inform the extent to which this effect is specific to the TADS-in manual rather than other aspects of inpatient treatment, it indicates that the manual was at least not associated with any negative effects. Birmaher et al. [7] report in their review that in the natural course, an episode of MDD has approximately a mean length of 7 to 9 months.

### Development of the TADS-in manual

The aim of this study was to pilot the newly adapted manual such that revisions can made before the manual is evaluated in a larger scale-controlled study. Weersing et al. [32] discuss the effectiveness of the CBT elements in manuals for the treatment of depression in adolescents in their review article. They identify the following elements: (1) basic psychoeducation, (2) pleasant activity scheduling and other behavioral activation techniques designed to directly raise mood, (3) cognitive restructuring strategies, (4) problem-solving skills training, and (5) other techniques (e.g., relaxation training, family therapy maneuver). Comparing three manuals (“coping with depression for adolescents,” “Pittsburgh cognitive therapy,” and TADS), the TADS manual is described by Weersing et al. as one which contains all relevant CBT techniques in a shorter time (12 vs. 16 weeks). The adaption of the manual for the inpatient setting excluded the five optional sessions and did not shorten any of the core elements. A question that is raised from Weersing et al. in this context is whether there is a “dose x technique minimum threshold for core components of CBT.” A meta-analysis of Weisz et al. [9] on the Effects of Psychotherapy for Depression in Children and Adolescents showed no correlation between treatment duration and outcome. In the meta-analysis by Weisz et al. [9], treatment duration was between 4 and 32 h with a mean of 13.5 and a median of 12 h. The TADS manual with 10 h is slightly below the average (included inpatient and outpatient treatment).

The experiences of clinicians delivering the intervention were collected in a focus group. Clinicians raised concerns about the transfer of learned strategies into the family environment being challenging when the therapy took place in an inpatient environment. Future adaptations of the manual might include the clinician devoting time to specifically address this problem. A related suggestion was to rename the “homework” to “exercises” to improve patient adherence, since the majority of patients had not regularly completed homework.

Feedback from the focus group also indicated that the TADS-in treatment schedule was often too tight to address the complex issues that patients had. One solution might be to cut or shorten the introductory contents which patients may already be familiar with to leave room for more complex contents of TADS-in. An alternative solution would be to deliver two TADS-in sessions per week and more explicitly try to incorporate some of their additional issues (e.g., discharge planning and school change) into the TADS-in techniques learnt.

Some contents of the manual were rated by the therapists as particularly helpful (e.g., material from the session on cognitive restructuring). Clinicians also felt that parents and their relationship to their children benefitted more from the manual than they would do with treatment as usual. Although the session with the parents was the one most often missed, when parents did attend the manual provided them with a good framework for understanding the techniques, patients were intending to apply following discharge.

### Future studies

Before conducting a randomized controlled trial of a novel treatment manual, it is important to check the feasibility in a pilot study. By piloting the manual in an inpatient setting, we were able to make numerous discoveries which could improve the planning and conducting of future controlled trials of the manual. We had some problems regarding the return of questionnaires due to the high clinical workload of the therapists delivering the intervention. For example, an interesting question would be which effect each session had on the outcome measures. Unfortunately, the weekly assessment was rarely completed and these data cannot therefore be evaluated. In future trials, more resources would need to be allocated for study organization and monitoring. Sufficient time should also be allocated for therapists to complete the study documentation more reliably (treatment fidelity data were missing for 5/29 patients). Due to limited resources, it was not possible to videotape the sessions to determine treatment fidelity. Instead, clinicians reported themselves how much of the manual they had been able to administer. These self-reports are of course open to bias and should be avoided in future studies. Also, sufficient resources need to be invested in dealing with informed consent and inclusion criteria assessment if sufficient time is to be available for delivering the treatment before a patient is discharged. Furthermore, future studies should carefully consider their outcome measures. In future studies, we would recommend ensuring that external reports of symptomology are provided by the same member of the multi-disciplinary team. Our qualitative analysis suggested that parent-child interactions were improved through the manual so future studies may seek to include this as an outcome measure.

Although interviews with clinicians using the manual identified numerous aspects which were not obtained in the quantitative evaluation, we did not have the resources to conduct interviews with the patients themselves. This could be a useful approach in future evaluations of the manual.

Future trials should also consider issues of implementation such as scaling of the intervention and care packages that should come after the intervention. Of course, given international differences in healthcare systems, these might vary between countries.

Finally, future large-scale studies might consider exploring the potentially moderating role of various factors. Clinicians felt that comorbidity with non-affective disorders (e.g., eating disorders, attention deficit disorders), cognitive or social impairment, previous experience with psychotherapy including CBT, a preference for structure, reluctance to engage with the therapist, and/or motivation for treatment all impacted on how much a patient benefited from the TADS-in manual. Whether this really is the case, it can only be investigated by standardizing assessment of these factors and administering measures prior to delivering the intervention.

Due to the heavy and diverse workload of clinicians working in the inpatient setting, a lot of personnel resources are needed to ensure prompt consent, the return of questionnaires, and for study documentation. In addition, the temporary length of stay, which sometimes does not match the number of sessions in the manual, was a problem in this pilot study. As a result, many patients could not be included in the analysis due to early discharge. Since the test-retest reliability of the FBB-DES was low, improved training may be needed in future trials using external reports. However, the self-assessment by the BDI proved to be useful as a pre-post measurement. Ideally, a trial in this area should be a blinded (in terms of external assessment) and randomized. It would be good to have a comparison group with treatment as usual. For future studies, it would also make sense to include and evaluate data on the pretreatment and duration of the depressive episode before the start of treatment. Future trials should also consider issues of implementation such as scaling of the intervention and care packages that should come after the intervention. Of course, given international differences in healthcare systems, these might vary between countries.

### Strengths and limitations of the study

There are some limitations to the current study which are worth acknowledging. The first relates to the sample size and generalizability of the study findings. Of the 29 patients enrolled in the study, only 19 remained at the end of the 8-week treatment. Although the modest sample size did not prevent us from observing a significant improvement in symptoms following the intervention, the relatively high dropout rate limits the generalizability of the findings. Nevertheless, it is hard to say whether our effects are likely to be over- or underestimates of the true effect. Dropout from the study was largely due to the fact that patients were discharged before all ten sessions could be completed. This was mainly caused by the length of time it often took to obtain informed consent from the patient and their parent(s) and conduct assessments to determine eligibility for the study. While this contributed to the smaller sample size, it is unlikely to cause a bias in the size of the effect found. Other patients were discharged from the clinic before completing the study because their symptoms had reduced to such an extent that they no longer required inpatient treatment. In this case, our effect sizes may be an underestimate of the true effect of the manual. However, other patients may have failed to complete the treatment because they or their therapist did not observe any improvements. In this case, effect sizes could be overestimates. A related limitation is that therapists were responsible for recruiting patients into the study themselves. As such, it is possible that they selected patients who they believed could benefit from the manual which also compromises generalizability. Future studies will be able to address this limitation through the recruitment of a larger sample size and by investing in resources to ensure patients receive the intervention as soon after admission as possible.

A second limitation is the lack of a control group, which is a necessary next step if the positive treatment effects are to be attributed to the TADS-in manual specifically. While a control group would have improved the robustness of our findings, the primary aim of the study was to pilot the novel intervention without incurring the increased costs which would have been associated with collecting data from twice as many patients. Relatedly, a follow-up data collection point would have been desirable to determine the longevity of the effects found. This would be an important aspect of study design in any future controlled studies of the manual.

Another limitation is that in our study, we did not collect data on minimally important differences (MID). In future larger studies, however, this would be recommended in order to have an indication of the effectiveness of the treatment from the view of the patient.

Finally, not all therapists were equally trained in the use of CBT and therefore had different expertise, which could have affected our findings. Some therapists in the focus group with a psychodynamic background said that working with the manual felt strange but that they did not think it had impacted treatment outcomes. Nevertheless, it is possible that the effects found would have been even greater if all therapists had been trained in using CBT. Information about treatment fidelity suggests not only that participants completed a relatively high proportion of sessions, but also that the therapists delivered the content for the sessions with a high level of fidelity.

Despite these limitations, the study also carries various strengths. The current study makes a significant contribution to the literature by presenting the first manual for the inpatient individual treatment of adolescent depression. Manualization of psychotherapeutic approaches is important not only for maximizing integrity and uniformity of therapy in treatment evaluation [33] but also for the dissemination and implementation of the treatment into practice [34]. Furthermore, the temporally limited and clearly structured nature of treatment manuals mean that they are often more cost-efficient, teachable, and conveyable [35]. We highlighted numerous differences between the inpatient and outpatient setting which make it inappropriate to assume that existing CBT manuals developed for the outpatient setting will also be effective in the inpatient setting.

A second strength of the study is that the outcome variables were obtained from various informants (patient self-report, healthcare assistant, clinician, clinical interview). Furthermore, we used administered clinically meaningful outcome measures such as clinical interviews to assess changes in diagnostic status. Being able to demonstrate positive effects of the intervention on diagnostic status assessed by a member of the research team who was not involved in delivering the intervention further strengthens the study findings.

A final strength of the study is the combination of quantitative and qualitative methods. Mixed-methods approaches are becoming increasingly common in the evaluation of psychotherapy [36–38] and depression treatments specifically [39–43]. Yet, few studies have applied mixed-methods approaches in the evaluation of treatment interventions for adolescent depression. They enable not only the testing of a priori hypotheses but also the discovery of unexpected findings.

## Conclusion

In summary, the current study presents the first evaluation of a promising manual for the individual inpatient treatment of adolescent depression (TADS-in). Preliminary data suggest patients showed clinical improvements during treatment similar to those found in the TADS study of outpatient treatment. Feedback from therapists using the manual could be used to further develop it before future controlled studies investigate whether the TADS-in manual shows superior effects to treatment as usual.

## Supplementary information

**Additional file 1.** An example of a checklist used to determine treatment fidelity.

**Additional file 2.** The interview schedule for the focus group

## Data Availability

Anonymized data are available on the Open Science Framework; https://osf.io/mnwb4/?view_only=350969c05ec24326b7173cbf7701b478

## Abbreviations

CBT: Cognitive behavioral therapy
TADS: Treatment of adolescent depression
TADS-in: Treatment of adolescent depression for inpatient setting
MDD: Major depressive disorder
MDE: Major depressive episode
ICD-10: International Statistical Classification of Diseases and Related Health Problems
DSM-IV: Diagnostic and Statistical Manual of Mental Disorders
BDI: Beck Depression Inventory
CGI: Clinical Global Impression
CGI-S: Clinical Global Impression—severity of psychopathology
CGI-I: Clinical Global Impression—change from the initiation of treatment
RCT: Randomized controlled trial
IPT-A: Interpersonal therapy for adolescents
CWD-A: Coping with depression—adolescents
Kinder-DIPS: Diagnostic Interview of Mental Disorders in Children and Adolescents
CFT: Culture Fair Intelligence Test
DISYPS: Diagnostic System for Mental Disorders in Childhood and Adolescence
FBB-DES: Fremdbeurteilungsbogen Depression (Depression Rating Scale of DISYPS-II)
SD: Standard deviation
ES: Effect size
SPSS: Statistical Package for the Social Sciences

## Footnotes

1 Oxford Centre for evidence-based medicine 2011, levels of evidence

2 Effect size was calculated using the formula (meanpost−meanpre)/SDdiff.

3 Effect size was calculated using the formula (meanpre−meanpost)/SDdiff.

4 Effect size was calculated using the formula (meanpre−meanpost)/SDdiff.

5 The number given here reflects the number of focus group participants who explicitly provided endorsement of a statement. The number of consenting participants (nods of agreement) is not shown here.
