# Supplementary material for: The adaptation and evaluation of a CBT-based manual for the inpatient treatment of youth depression: a pilot study
Source: Pilot Feasibility Stud. 2020 Feb 24;6:30. doi: 10.1186/s40814-020-00573-6 (PMC7038626; doi:10.1186/s40814-020-00573-6)
Supplement: Supplementary file 1 — Additional file 1. An example of a checklist used to determine treatment fidelity. [file 40814_2020_573_MOESM1_ESM.docx]

**1. Checklist Treatment model and setting goals**

Date: ________________________________

| Topics | Done? |
| --- | --- |
| 1. Become acquainted with each other and go over the treatment plan |  |
| 1. Short overview of the main outcomes of the diagnostic phase and how they relate to the model of depression |  |
| 1. Clarify the procedure of cognitive-behavioural therapy, why it is important to involve the whole family and to what extent the treatment can be helpful for the adolescent |  |
| 1. Clarify the parents‘ and adolescents‘ goals for treatment |  |
| 1. No-suicide contract and answering questions about treatment |  |
