# Supplementary material for: The adaptation and evaluation of a CBT-based manual for the inpatient treatment of youth depression: a pilot study
Source: Pilot Feasibility Stud. 2020 Feb 24;6:30. doi: 10.1186/s40814-020-00573-6 (PMC7038626; doi:10.1186/s40814-020-00573-6)
Supplement: Supplementary file 2 — Additional file 2. The interview schedule for the focus group [file 40814_2020_573_MOESM2_ESM.docx]

**The TADS-in pilot study**

**Focus Group Interview Schedule**

Thank you for your support in implementing the TADS-in study and your willingness to participate in the qualitative focus group today! In addition the data we have already collected about the TADS-in manual, we would like to **survey** therapists’ opinions concerning the feasibility, the content and the acceptance of the manual. We will use this information for publication of the study. The pilot study will help to inform a randomized controlled trial about the effectiveness of a CBT-based treatment intervention in a larger sample.

**If you agree** to take part we will record the session with a dictaphone in order to transcribe the discussion later. No names will be mentioned in the transcription. First of all we have some basic rules that should be **observed**:

- Everyone can answer freely to the questions with whatever comes to his/her mind, without a fixed order. However, to avoid missing important feedback, please talk one after another.
- There are no right or wrong answers - we just want to hear your honest opinion.

**Questions/Topics:**

*General set-up*

1. Did the frequency of conducting a TADS-in session every second individual therapy session **prove successful**?
2. Did the content of the manual match the timeframe?
3. Was the number of sessions enough?
4. How compatible was the content of the manual with other elements of inpatient therapy?
   1. Were there aspects of the inpatient setting which were particularly beneficial for the manual (e.g. weekly goals which supported the patients’ realisation of homework)?
   2. Were there any aspects of the inpatient setting which were obstructive for the manual (e.g. overlapping content or different goals)?
5. Were there any organisational problems with delivering the manual (templates not available, instructions incomprehensible, missing contact person)?
6. Do you have other suggestions concerning the general set-up?

*Target group*

1. Which patients were particularly interested in the TADS-in treatment?
2. Which patients were less motivated for the TADS-in treatment?
   1. How did you handle this?
3. How did the parents react to the TADS-in manual during their sessions?
4. Did parents and children discuss contents of the manual?
5. Do you have other suggestions concerning the target group?

*Content*

1. Were there elements of the manual which you found particularly helpful?
2. Were there elements of the manual which were difficult to implement?
3. Did parent-child interactions benefit from TADS-in?
4. What were frequent topics in the non-TADS-in therapy sessions?
5. What were reasons for **terminating** the TADS-in treatment?
6. Do you have other suggestions concerning the content of the manual?

*Therapist*

1. Did you as practitioner enjoy working with the TADS-manual?
2. Did the orientation of your clinical training play a role in working with the manual?
3. What is your experience of working with other therapy manuals?
4. In which situations was it difficult to implement the TADS-in manual?
5. In which situations did you experience it as particularly helpful?
6. Do you have other suggestions from a therapist’s point of view?
7. Is there anything that we haven’t yet asked but what you would like to add?

Thank you for your help!
